# Mint Oil, ɤ-Tocopherol, and Whole Yeast Cell in Sow Diets Enhance Offspring Performance in the Postweaning Period

**DOI:** 10.3389/fvets.2021.658956

**Published:** 2021-07-12

**Authors:** Lily P. Hernandez, James L. Dunn, Joel Wenninghoff, Amanda Hesse, Crystal L. Levesque

**Affiliations:** ^1^Department of Animal Science, South Dakota State University, Brookings, SD, United States; ^2^Archer Daniels Midland Animal Nutrition, Quincy, IL, United States

**Keywords:** sows, oxidative stress, lactation, gestation, offspring, performance

## Abstract

Times of high metabolic activity in gestation and lactation, as well as periods of stress at weaning, can lead to greater incidences of oxidative stress in the dam and offspring during the suckling and postweaning period. Oxidative stress is an imbalance between prooxidant molecules and the antioxidant defense system that can negatively impact growth and/or reproductive performance. The objective of this research was to evaluate the effectiveness of whole yeast cell, peppermint oil, and ɤ-tocopherol in gestation and lactation on maternal oxidative status and offspring growth from birth to market. In study 1, 45 sows and gilts were assigned to one of four diets [control diet (CON), control + whole yeast cell (YC), control + mint oil top dress (MO), and control + yeast cell and mint oil top dress (YCMO)] provided from d110 of gestation through to weaning. A total of 481 weaned offspring were randomly allotted to pens balanced by weight and litter within maternal treatment and received the same dietary treatment as the sow for 35 days postwean in a four-phase feeding regimen. In study 2, 53 sows and gilts were allotted to four diet regimens similar to study 1 [CON, YC, MO, and control + ɤ-tocopherol (GT)] from d5 postbreeding to weaning. At weaning, 605 piglets were randomly allotted to pens, balanced by weight and litter within maternal treatment and fed a common diet for 126 days postwean in a nine-phase feeding regimen. Maternal dietary treatment did not impact sow body weight, piglet birth weight, and litter size in either study. In study 1, piglets from YC sows were heavier (*p* < 0.05) at weaning than CON animals. In the postwean period, overall daily gain was greater (*p* < 0.05) for CON-fed pigs than YCMO pigs, with overall feed intake greater (*p* < 0.05) for YCMO- than MO-fed pigs, resulting in lower (*p* < 0.05) Gain to Feed (G:F) in YCMO-fed pigs. In study 1, glutathione content in milk tended to be lower (*p* < 0.10) in MO than in YCMO sows. In study 2, piglets from GT-fed sows tended to be heavier (*p* < 0.10) at weaning than YC piglets. Lightweight pigs from CON sows tended to be lighter (*p* < 0.10) than pigs from all other treatment groups at weaning and day (d) 29 postwean. Lightweight MO and GT pigs were heavier at d42 (*p* < 0.05) than CON and YC pigs. At d70 postwean, GT pigs tended to be heavier than CON pigs. Lightweight MO pigs had greater gain (*p* < 0.05) during the finishing period than all other treatment groups. With respect to sow oxidative status in study 2, glutathione content in colostrum and d4 and 14 milk samples did not differ by maternal treatment. Superoxide dismutase activity in sow sera, colostrum, and milk did not differ between diets in either study. Whole yeast cell and ɤ-tocopherol supplementation in sow lactation diets resulted in heavier offspring. However, pre- and postnatal exposure to mint oil benefited lightweight pigs up to market weight.

## Introduction

Swine industry performance as a whole relies ultimately on the efficiency of the sow. In this regard, genetic selection over the past decades has resulted in highly prolific females and the production of lean progeny (1). Modern sows are farrowing more than 15 total piglets born, and the average number of pigs weaned per sow per year appears destined to routinely surpass 30 pigs weaned/sow over the next few years. There are, however, negative implications and new challenges that arise with this advancement. These include higher incidences of low-birth-weight piglets, increased within-litter weight variability, and increased preweaning mortality (2, 3). In this context, the productivity indicators that reflect the quality of piglets produced as well as the number of piglets, such as kilograms of pork per sow per year, may better reflect advancement in productivity than simply pigs produced per sow per year (4).

Increased number of piglets per litter ultimately results in an increased demand on the sow to meet the nutritional needs of her offspring. Late gestation and the lactation period are noted points where the sow mobilizes body tissues for nutrients to maintain fetal growth (5, 6) and offspring postnatal growth, particularly in early lactation where daily feed intake is insufficient to meet milk yield (7, 8). When mobilization occurs for an extended period of time, oxidative stress increases. Oxidative stress is defined as the imbalance between prooxidants and antioxidants, with conditions that favor the production of prooxidants (9), which can arise from changes in metabolism or immune status of an animal. In the livestock industry, oxidative stress has been recognized as one of the major threats to animal welfare, productive performance, and quality animal products (10–12). For the gestating and lactating dam, diminished performance is less than desirable as it can impact both sow, and offspring performance. Weaning is also a time of stress for young animals and has been linked to greater incidence of oxidative stress. If present, performance of the sow and her offspring, pre- and postwean, is reported to be reduced (1, 13).

Inclusion of yeast-based products and antioxidant-rich ingredients, such as phytochemical oils or vitamins, in sow diets is reported to positively alter antioxidant status of the dam (14–17). However, there is little information on the potential carryover effects of the inclusion of antioxidant-rich feed additives in gestation and lactation diets on offspring postweaning performance.

Peppermint oil is a hybrid species of mint, coming from the breeding of spearmint and water mint (18) in the *Mentha* genus and is known to be rich in antioxidant activity. As oxidative stress is involved in the relationship between the production of antioxidants and free radicals, the presence of a feed additive that could supply exogenous antioxidants to the sow may be beneficial in reducing an elevated oxidative status. There is little research evaluating the impact of certain strains of yeast on the reduction in free radicals in swine or even alleviating the negative effects caused by oxidative stress. An inactive whole yeast cell product, derived from *Pichia guilliermondii* (CitriStim, ADM Alliance Nutrition), has been linked with modulating immune functions (19). It is possible that modifying certain functions of the immune system could aid in reducing oxidative stress in the female or her offspring. Vitamin E is also known for its antioxidant activity. Derivatives of vitamin E are also found to possess antioxidant activity in their chemical makeup, although, the antioxidant capacity of the different stereoisomers does vary (20). Γ-tocopherol, RRR stereoisomer and natural source, is a lesser-known tocopherol derivative of vitamin E. Although, there have been multiplies studies that have deemed ɤ-tocopherol effective against oxidative stress (21–25), little have investigated the stereoisomer's success in swine or the use of a natural source of ɤ-tocopherol in diets. However, if similar to α-tocopherol, which has the most effective antioxidant capacity of all the stereoisomers of vitamin E (20), this source may have potential in mitigating the negative effects of oxidative stress in pigs.

The objective of these studies was to observe the impacts of including a whole yeast cell component, a phytogenic oil (peppermint oil), and a vitamin E stereoisomer (γ-tocopherol) solely in sow and nursery pig diets on sow antioxidant capacity and offspring performance. It was hypothesized that the inclusion of the feed ingredients would result in the reduction in oxidative stress during high points of metabolic activity, such as late gestation and lactation, in turn mitigating negative effects that impact dam performance and ultimately improve offspring performance during the suckling and the postweaning period.

## Materials and Methods

### Animals, Management, and Experimental Procedure

Two separate studies were conducted to accomplish the overall objective. Study 1 utilized whole yeast cell and phytogenic oil alone or in combination included during the lactation period. Based on the lack of additive effect observed in study 1 and to assess the impact of longer maternal supplementation period, study 2 assessed maternal supplementation of whole yeast cell, phytogenic oil, or vitamin E stereoisomer 5 days following breeding through lactation.

#### Study 1

Forty-five multiparous and primiparous sows (PIC 1050; 240.71 ± 38.51 kg), across two blocks, were randomly allotted at day (d) 110 of gestation to one of four experimental diets provided in mash form (*n* = 10–12 animals/treatment) in a 2 × 2 factorial treatment design balanced by body weight (BW), backfat (BF), and parity. Dietary treatments were as follows: control (CON), whole yeast cell (YC), mint oil (MO), and whole yeast cell + mint oil (YCMO). Control was a standard lactation diet formulated to meet or exceed nutrient requirements for sows in accordance with the National Research Council (NRC) (2012; Table 1). Whole yeast cell (CitriStim, ADM Animal Nutrition; Quincy, IL) was added at 0.2% based on the manufacturer's instructions at the expense of corn to the control diet. A top dress of either mint oil or a carrier was provided at 50 g/day (set to ensure 10 ppm of active peppermint oil/day) and placed on top of the 0800 h feeding. Sows and gilts were moved into the farrowing room ~5 days prior to parturition and housed in individual farrowing crates (1.83 × 2.43 m). Feed was dispensed by an electronic feeding system (Gestal 3G; Jyga Technologies, Greenlu, KS, USA), allowing daily intake up to 20% above the curve set to achieve *ad libitum* intake by 5 days of lactation. Females were assigned to parity-based feed curves (i.e., parity 1, parity 2, parity 3+); daily intake targets within each curve are based on historical barn lactation intake. Water was provided *ad libitum*. Sows and gilts were supervised 24 h/day from birth of the first piglet to the last piglet born in the farrowing group; farrowing was completed over ~5 days. Oxytocin (Aspen Veterinary Resources, Liberty, MO, USA) was administered on an “as needed” basis [i.e., prolonged farrowing (>8 h), birth interval >90 min, and >10 piglets born, >2 piglets pulled]. Sows receiving oxytocin represented <20% of all females. Piglets were managed as per standard barn protocols for vaccinations, iron injections, tail docking, and castration in that a 2-ml intramuscular (i.m.) injection of iron dextran (Uniferon 200, Pharmacosmos, Watchung, NJ, USA), 1 ml oral dosage of ponazuril (Marquis, Merial, Duluth, GA, USA), and, if birth weight was under 1 kg, a 0.25-ml i.m. injection of Excede (Zoetis, Pasippany, NJ, USA) at d1 of lactation were given. At 3d postfarrowing, tail docking and castration were performed, and piglets were administered a 1-ml i.m. injection of Circumvent PCV-M G2 (Merck Animal Health, Madison, NJ, USA). Two weeks following the completion of farrowing, all piglets were orally vaccinated with a 1-ml dose of *Escherichia coli* vaccine (Entero-vac, ARKO Laboratories, Jewell, IA, USA), and at weaning, all animals were administered a 1-ml i.m. injection of enrofloxacin (Baytril 100, Bayer, Shawnee Mission, KS, USA) and Circumvent PCV-M G2. Litters were equalized to 12 to 14 pigs within 48 h by means of cross fostering or removal within maternal treatment groups. Removals included piglets that were deemed to be runts (≤600 g at birth) and fallbacks [average daily gain (ADG) of ≤30 g] that were taken off test and artificially reared on milk replacer (Birthright Baby Pig Milk Replacer, Ralco, Marshall, MN, USA) using milk decks (Birthright Milk Deck, Ralco, Marshall, MN, USA).

**Table 1:** Ingredient composition and nutrient content of sow diets in studies 1 (lactation) and 2 (gestation/lactation).

**Ingredients %**	**Study 1^1^**		**Study 2^2^**	
	**Control**	**YC**	**Gestation**	**Lactation**
Corn	66.27	66.07	81.61	66.47
Soybean meal, 46.5%	29.85	29.85	14.54	29.85
Monocalcium phosphate	1.76	1.76	1.84	1.76
Calcium carbonate	1.22	1.22	1.31	1.22
Salt	0.5	0.5	0.50	0.50
Sow vitamin premix^3^	0.05	0.05	0.05	0.05
Trace mineral premix^4^	0.15	0.15	0.15	0.15
Toxin binder^5^	-	-	0.20	0.20
Whole yeast cell	0.0	0.2	-	-
Calculated nutrient content, %				
Dry matter	87.61	87.61	89.50	89.60
Crude protein	18.58	18.58	13.5	19.40
Crude fat	2.51	2.51	2.50	2.80
ME, kcal/kg	3278.3	3278.3	3284.9	3276.1
Calcium	0.92	0.92	0.89	0.89
Phosphorus	0.70	0.70	0.72	0.76
Phos avail-swine	0.45	0.45	0.44	0.44
SID lysine	1.06	1.06	0.55	0.97
Methionine: lysine	0.29	0.29	0.38	0.29
Thrionine: lysine	0.64	0.64	0.76	0.64
Tryptophan: lysine	0.21	0.21	0.22	0.21
Analyzed nutrient content, %				
Dry matter	86.73	86.57	86.74	86.73
Crude protein	18.66	21.44	12.69	18.33
Crude fat	1.39	1.44	1.78	1.72
Total Lys	1.10	1.24	0.73	1.20

1 *Peppermint oil (MO) at 50 g/day was added as a top dress to provide 10 ppm of active peppermint oil/day in a 2 × 2 factorial with or without whole yeast cell (YC, CitriStim, ADM Animal Nutrition, Quincy, IL) and with or without MO. Diets were provided to pregnant females from entry to farrowing room (d110 of gestation) through weaning*.

2 *Whole yeast cell was included in the diet at 0.20%, and mint oil was added as a top dress at 50 g/day to provide 10 ppm mint oil/day in lactation (study 1). In each of gestation and lactation (study 2), additives were top dressed at 50 g/day to supply 3.1 g whole yeast cell, 10 ppm mint oil, and 200 ppm ɤ-tocopherol*.

3 *Provided vitamins A (9,992,000 IU/lb), D3 (1,499,000 IU/lb), E (50,000 IU/lb), B12 (40 mg/lb), menadione (4,000 mg/lb), riboflavin (9,000 mg/lb), D-pantothenic acid (55,000 mg/lb), niacin (50,000 mg/lb), folic acid (1,000 mg/lb), pyridoxine (3,000 mg/lb), thiamine (3,000 mg/lb), and biotin (155 mg/lb)*.

4 *Provided copper (1.10%), manganese (2.94%), selenium (200 ppm), and zinc (11%)*.

5 *Toxin binder was not added in study 1. Toxin binder that was utilized for study 2 was AncoFit (ADM Animal Nutrition, Quincy, IL, USA)*.

At day 21 ± 2 of lactation, all piglets were weaned and transferred to the South Dakota State University wean-to-finish barn. Offspring that were reared on the sow for the duration of lactation were allotted to pens within maternal treatment (six to eight pigs/pen; 16–17 pens/maternal treatment at 0.53 m^2^/pig; 481 total pigs; 6.25 ± 3.35 kg) and received the same dietary treatment as their dam during lactation. To differentiate maternal diets from nursery diets, postweaning dietary treatments were given the subscript “wean” (e.g., YC_wean_). Pens were balanced for weight and litter as much as possible, ensuring that no more than two pigs from the same litter were in a pen to keep potential social stress at weaning as similar among pens of pigs as possible. Within a four-phase nursery pig feeding program, all diets were formulated to meet nutrient requirements of weaned pigs according to the NRC (2012). Dietary inclusion level of whole yeast cell was the same in lactation, with both mint oil and carrier included as a fat blend mix at 0.1% of the diet. Phases 1 and 2 were provided in pellet form with phases 3 and 4 as meal (Table 2). Water was provided *ad libitum*.

**Table 2:** Ingredient composition and nutrient content of the control weaned pig diets in studies 1 and 2.

**Ingredients, %**	**Study 1^1^**				**Study 2^2^^,^^6^**				
	**Phase 1**	**Phase 2**	**Phase 3**	**Phase 4**	**Phase 5**	**Phase 6**	**Phase 7**	**Phase 8**	**Phase 9**
Corn	30.15	35.72	54.20	69.96	72.96	79.25	83.30	86.54	90.15
Spray dried whey	30.00	25.00	10.00	-	-	-	-	-	-
Soybean meal, 46.5%	17.57	19.45	27.71	30.35	23.66	17.68	13.83	10.77	7.29
HP 300^3^	8.00	7.00	-	-	-	-	-	-	-
Menhaden fishmeal	8.00	7.00	3.00	-	-	-	-	-	-
Soy oil	4.30	3.70	2.00	-	-	-	-	-	-
Monocalcium phosphate	0.44	0.50	1.10	1.41	1.21	0.98	0.85	0.76	0.68
Zinc oxide	0.42	0.42	0.28	-	-	-	-	-	-
Calcium carbonate	0.25	0.32	0.85	1.02	1.02	0.97	0.94	0.91	0.88
Lysine-HCl	0.25	0.26	0.32	0.40	0.40	0.40	0.38	0.36	0.34
Methionine	0.20	0.20	0.15	0.15	0.11	0.08	0.07	0.04	0.03
Threonine	0.11	0.11	0.12	0.15	0.14	0.13	0.12	0.11	0.11
Tryptophan	0.03	0.03	0.03	–	–	0.01	0.01	0.01	0.02
Vitamin premix^4^	0.05	0.05	0.05	0.05	0.05	0.05	0.05	0.05	0.05
Mineral premix^5^	0.15	0.15	0.15	0.15	0.15	0.15	0.15	0.15	0.15
Selenium	0.05	0.05	–	–	-	–	–	–	–
Salt	0.03	0.03	0.03	0.35	0.30	0.30	0.30	0.30	0.30
C18:fat base	0.01	0.01	0.01	0.01	–	–	–	–	–
Calculated nutrient content, %									
DM	91.14	90.58	88.78	87.62	89.50	89.50	89.40	89.40	89.30
CP	23.85	23.34	20.30	19.35	19.60	15.30	3.90	12.70	11.30
Lysine, SID	1.77	1.71	1.46	1.38	1.08	0.94	0.84	0.75	0.65
Analyzed nutrient content, %									
Dry matter	91.80	92.22	88.98	81.35	87.24	87.02	86.40	87.31	86.89
Crude protein	24.33	23.64	21.32	18.87	17.09	14.02	12.00	10.86	12.89
Total lysine	1.69	1.74	1.57	1.42	1.11	0.83	0.66	0.62	0.74

1 *In each phase, whole yeast cell component (CitriStim, ADM Animal Nutrition, Quincy, IL) was added to the control diet at 0.2% at the expense of corn to create the YC_wean_ diets; peppermint oil was added at 0.01% to the control and YC_wean_ diets to create MO_wean_ and YCMO_wean_ diets. The C:18 fat base was included in the control and YC_wean_ diets at 0.01% to balance fat inclusion across all treatments*.

2 *The whole yeast cell, mint oil, and γ-tocopherol were top dressed at 50 g/day and supplied 3.1 g whole yeast cell/day, 10 ppm active peppermint oil/day, and 200 ppm γ-tocopherol/day, respectively*.

3 *HP300, Hamlet Protein, Findlay, OH*.

4 *Provided vitamins A (9,992,000 IU/lb), D3 (1,499,000 IU/lb), E (50,000 IU/lb), B12 (40 mg/lb), menadione (4,000 mg/lb), riboflavin (9,000 mg/lb), D-pantothenic acid (55,000 mg/lb), niacin (50,000 mg/lb), folic acid (1,000 mg/lb), pyridoxine (3,000 mg/lb), thiamine (3,000 mg/lb), and biotin (155 mg/lb)*.

5 *Provided copper (1.10%), manganese (2.94%), selenium (200 ppm), and zinc (11%)*.

6 *Phases 3 and 4 diet formulations were the same as that reported in study 1; thus, they were not reported under study 2 to reduce redundancy*.

#### Study 2

Fifty-three sows and gilts (PIC 1050; 206.21 ± 35.26 kg), in two blocks, were randomly assigned to one of four experimental diets at breeding (*n* = 12–14 animals/treatment) balanced by BW, BF, and parity. Females were housed in gestation stalls (0.61 × 1.98 m) from breeding to d110 of gestation. Dietary treatments were as follows and similar to that reported for study 1: CON, YC, MO, and standard diet with γ-tocopherol (GT). The whole yeast cell, mint oil, and γ-tocopherol were top dressed at 50 g/day and supplied 3.1 g whole yeast cell/day, 10 ppm active peppermint oil/day, and 200 ppm γ-tocopherol/day, respectively. All top dressing was completed at the 0600 h feeding during gestation and 0800 h feeding during lactation. Application of top dress started at 25% of calculated volume at d2 of gestation and increased daily such that the experimental period began at d5 of breeding through weaning. Gestation and lactation diets were formulated to meet or exceed nutrient requirements for sows according to the NRC (2012; Table 3) and provided in mash form. Amount of feed allotted in gestation was determined based on body condition score (BCS), with allocation in farrowing based on parity. Water was provided *ad libitum* during both periods. Movement to farrowing rooms and daily sow and litter management were the same as that described for study 1. As noted in study 1, fallbacks and runts were removed from the sow and artificially reared on milk replacer throughout the lactation period.

**Table 3:** Reproductive performance, sow antioxidant status, and suckling pig performance from sows provided lactation diets with or without a whole yeast cell component (YC) and with or without peppermint oil (MO) top dress (study 1)^1^^,^^4^^,^^5^.

**Items**	**CON**	**YC**	**MO**	**YCMO**	**SEM**	***p*-value**
No. sows/litter	12^2^	11	11	11^2^		
Sow lactation feed intake, kg/day	6.41	6.59	6.68	6.64	0.42	0.952
Litter, average						
Born alive	14.08	13.82	14.82	13.82	0.87	0.831
Stillborn	0.92	0.72	0.91	1.27	0.33	0.706
Mummies	1.1	0.97	1.32	0.93	0.34	0.784
Weaned	10.91	10.02	11.48	10.03	0.93	0.325
% Removed^3^	18.9	20.4	16.6	24.3		
Piglet BW, kg						
Birth	1.46	1.44	1.39	1.39	0.05	0.729
Wean	6.45^b^	6.93^a^	6.71^ab^	6.56^ab^	0.16	0.013
kg weaned/sow	67.7	67.7	75.3	64.2	4.30	0.288
ADG, g/day, suckling	230^b^	240^a^	230^ab^	240^ab^	0.01	0.041
GSH, U/ml						
Colostrum	2.6	1.75	1.04	2.07	0.61	0.348
Milk, lactation d4	20.56^x^^,^^y^	21.13^x^^,^^y^	15.16^y^	32.05^x^	4.93	0.059
SOD in sow serum, U/ml						
D110 of gestation	7.16	10.05	10.12	5.66	4.07	0.751
Weaning	4.40	4.11	5.52	5.10	0.80	0.393

1 *Peppermint oil at 50 g/day was added as a top dress to provide 10 ppm of active peppermint oil/day in a 2 × 2 factorial with or without a whole yeast cell component (0.2% of diet, CitriStim, ADM Animal Nutrition, Quincy, IL) and with or without MO. Diets were provided to pregnant females from entry to farrowing room through weaning*.

2 *One sow in each of CON and YCMO and their litters were removed from the trial due to sow feed consumption issues in lactation and refusal to nurse piglets*.

3 *Calculated as all pigs removed (pigs that died and fallbacks/starveouts moved to birth decks) from a treatment group divided by total pigs born*.

4 *Values denoted with superscript*

x,y *indicate a tendency (p < 0.10)*.

5 *Values denoted with superscript*

a,b,c,d *indicate a significance (p < 0.05)*.

At day 21 ± 2 of age, pigs were weaned. Because of limited finishing space, pigs from block 1 were weaned to a research farm located outside of Brookings, SD (off-site; 13–14 pigs/pen; 0.40 m^2^/pig), and pigs from block 2 were weaned to the South Dakota State University wean-to-finish barn (on-site; 7–9 pigs/pen; 0.47 m^2^/pig) within maternal treatment. Across both blocks, a total of 605 pigs were weaned at 6.14 ± 2.53 kg (11–19 pens/maternal treatment) and balanced for weight and litter within pen as possible, ensuring no more than 3 pigs from the same litter were in a pen to keep potential social stress at weaning as similar among pens of pigs as possible. Pen stocking density was reduced (five to six pigs/pen) at d29 and d42 after weaning in blocks 1 and 2, respectively. To more specifically evaluate noted differences in light- and heavyweight offspring response to dietary treatment in study 1, those pigs deemed lightweight (<1.10 kg) and heavyweight (>1.65 kg) at birth were retained and followed through to market. Thus, pigs selected for removal to reduce stocking density were those deemed average weight (5.15–7.02 kg) at weaning. Offspring were fed a common diet throughout the postwean period. All diets were formulated to meet the nutrient requirements of weaned pigs within a nine-phase feeding program (Table 4). Phases 1 and 2 were provided in pellet form with all following phases provided as meal; paylean (dietary inclusion was 0.2%, Elanco, Greenfield, IN, USA) was added to phase 9 at 4.5–9 g/ton for 28 days. Water was provided *ad libitum*.

**Table 4:** Performance of weaned pigs provided diets with or without a whole yeast cell component (YC) and with or without a peppermint oil (MO) blend over a four-phase feeding program (study 1)^1^^,^^3^.

**Items**	**CON_**wean**_**	**YC_**wean**_**	**MO_**wean**_**	**YCMO_**wean**_**	**SEM**	***p*-value**
Body weight, kg						
Wean	6.45^b^	6.93^a^	6.71^ab^	6.56^ab^	0.16	0.013
d6	6.68^b^	7.12^a^	7.01^ba^	6.75^ab^	0.12	0.015
d13	8.97	9.07	9.26	9.02	0.16	0.478
d19	11.19	11.31	11.5	11.32	0.19	0.636
d35	19.78	19.8	19.17	19.96	0.35	0.303
Daily gain, kg/d^2^						
d0–d6	0.13^a^	0.12^ba^	0.11^abc^	0.09^c^	0.01	0.01
d6–d13	0.31	0.28	0.29	0.28	0.01	0.199
d13–d19	0.38	0.35	0.37	0.34	0.02	0.282
d19–d35	0.53	0.53	0.5	0.5	0.02	0.431
d0–d13	0.23^a^	0.20^ab^	0.21^ab^	0.19^b^	0.01	0.012
d0–d35	0.40^a^	0.39^ab^	0.38^ab^	0.37^b^	0.01	0.035
Daily feed intake, kg/d^2^						
d0–d6	0.13	0.13	0.13	0.12	0.01	0.23
d6–d13	0.29^abc^	0.30^b^	0.27^c^	0.30^ab^	0.01	0.002
d13–d19	0.39^b^	0.45^b^	0.45^ab^	0.53^a^	0.02	0.0001
d19–d35	1.08^a^	1.06^ab^	0.99^b^	1.08^ab^	0.04	0.032
d0–d13	0.22^a^	0.22^a^	0.20^b^	0.22^ab^	0.005	0.026
d0–d35	0.65^ab^	0.66^ab^	0.61^b^	0.69^a^	0.02	0.005
Gain: feed^2^						
d0–d6^4^	0.91	0.94	0.90	0.76	0.07	0.056
d6–d13	1.07^abc^	0.91^cb^	1.11^a^	0.92^b^	0.06	0.005
d13–d19	0.98^a^	0.79^ab^	0.82^ab^	0.63^b^	0.06	0.0006
d19–d35	0.5	0.5	0.51	0.46	0.02	0.448
d0–d13	1.03^a^	0.92^ab^	1.03^a^	0.88^b^	0.03	0.003
d0–d35	0.63^a^	0.59^ab^	0.62^a^	0.54^b^	0.02	0.018

1 *Weaned pigs were provided the same dietary treatment that had been applied to their respective dams during lactation. To differentiate the pre- and post-weaning period, treatments were defined as (CON_wean_, YC_wean_, MO_wean_, and YCMO_wean_, respectively). Phase 1 ran from d0 to d6 after weaning, phase 2 from d6 to d13, phase 3 from d13 to d19, and phase 4 from d19 to d35. Pigs were weaned at 21 ± 2 days of age. In each phase, a whole yeast cell component (CitriStim, ADM Animal Nutrition, Quincy, IL) was added to the control (CON) diet at 0.2% at the expense of corn to create the YC_wean_ diets; peppermint oil was added at 0.01% to the CON_wean_ and YC_wean_ diets to create MO_wean_ and YCMO_wean_ diets, respectively. The C:18 fat base was included in the CON_wean_ and YC_wean_ diets at 0.01% to balance fat inclusion across all treatments*.

2 *Calculated on a pen basis*.

3 *Values denoted with superscript*

a,b,c,d *indicate a significance (p < 0.05)*.

4 *Values presented appear to be incorrect; however, this may be in part due to the size of the feeders relative to weaned pig size. Feeders used for the study are designed to hold sufficient feed for finishing pigs, which is as much feed on a daily basis (i.e., 6 lb/finisher pig) as is budgeted for the entire first 7–10 days after weaning. Because of the size of the feeders, newly weaned pigs often use the feed pan area for sleeping or climb into the feeder to eat, which increases the risk of feed spillage into the pit. While all efforts are made to accurately account for feed disappearance in the first few days after weaning, what appears to be an error is a reflection of the mismatch between feed volume and feeder size*.

### Data Collection

#### Performance Values

In study 1, sow BW was recorded at d110 of gestation, within 24 h of parturition, and at weaning. For study 2, BW was recorded at breeding, d110 of gestation, within 24 h of parturition, and at weaning. Back fat at the last rib was measured at breeding, d110 of gestation, and weaning using an ultrasound (Ibex pro, E.I. Medical Imaging, Loveland, CO, USA). In lactation, feed orts were weighed and removed every third day. Litter characteristics at birth (total born, born alive, stillborn, mummies, and sex distribution) were recorded. Piglets were weighed within 24 h of farrowing at d4 and d7 of lactation and at weaning. Individual BW of weaned pigs was recorded at each phase through diet phase 4; in study 2, pigs were weighed monthly thereafter until market (~d129 after weaning). At each weigh period up to diet phase 4, BW was used to establish three weight categories (light, average, and heavy) with the use of quartiles. The upper limit of “lightweight” at birth was set as <1.1 kg based on an increased risk of preweaning mortality (26). Feed disappearance was recorded in tandem with weigh days. The ADG, average daily feed intake (ADFI), and G:F were calculated to determine pen performance.

#### Blood Sampling

Blood samples from the dam were collected *via* the jugular venipuncture into a non-heparinized blood collection tube (BD Vacutainer, Franklin Lakes, NJ, USA). Collections in study 1 occurred at d110 of gestation and weaning and at 3 days prior to breeding, d110 of gestation, and weaning in study 2. Blood samples were collected prior to feed drop in gestation and prior to the 0800 h feeding in lactation to ensure that feeding bouts were consistent across females for each collection. In study 2 on d2 of lactation beginning at 0800 h, a 1-ml blood sample was collected from the mammary vein of three piglets/litter (one piglet from each birth weight category). All samples were centrifuged at 28,000 × g for 20 min, and serum was collected and stored at −80°C until time of analysis.

#### Colostrum and Milk Sampling

In both studies, following the birth of the first piglet and prior to suckling, a 25-ml colostrum sample was collected from the dam *via* gentle stripping of all teats. At d4 and d14 of lactation, piglets were removed from the sow for 1 h followed by a 2-ml i.m. injection of oxytocic principle (Oxytocin, Aspen Veterinary Resources, Liberty, MO, USA) for milk collection (40 ml) from all teats. Piglets were returned to the dam following collections, and samples were stored at −20°C until further analysis.

### Indicators of Piglet Robustness

In study 2, piglet serum immunocrit at d2 of age was based on the methods of Vallet and Miles (27). Ideally, three pigs were chosen from each litter, but if no pigs fit a certain weight range, the observation for that category was then lost. In conjunction with piglet sera samples, immunocrit was evaluated in colostrum *via* a modified methodology from Vallet et al. (28). Colostrum samples were diluted in a 1:1 ratio with 10% bovine serum albumin in 0.9% saline. In duplicate, diluted colostrum samples were combined with 40% (wt/vol) ammonium sulfate in distilled water to precipitate immunoglobulins and then loaded into a hematocrit capillary tube and centrifuged at 12,000 × g for 10 min. Immunocrit ratio was determined as the ratio of the precipitate length divided by the total length of diluted colostrum, and then doubled to account for prior colostrum dilution.

Serum insulin-like growth factor 1 (IGF-1) was measured in sow serum at d110 of gestation as well as colostrum and milk samples. Concentration of IGF-1 was determined in duplicate by radioimmunoassay (29, 30) for blood, colostrum, and milk samples.

### Assessment of Dam Antioxidant Status

Superoxide dismutase (SOD) enzyme concentration in d110 of gestation and weaning sow sera were determined using a commercially available kit (Superoxide Dismutase Kit, Cayman Chemical, Ann Arbor, MI, USA), which utilized tetrazolium salt for detection of superoxide radicals generated by xanthine oxidase and hypoxanthine. Samples were analyzed in duplicate following the manufacturer's instructions. Colostrum and milk samples were analyzed following the manufacturer's instructions with the inclusion of a sample blank to correct for noise. Colostrum and milk glutathione (GSH) were assessed using a commercially available kit (Glutathione Assay Kit, Cayman Chemical, Ann Arbor, MI, USA). Samples were deproteinated prior to analysis in accordance with the manufacturer's instructions.

### Statistical Analysis of Results

Reproductive performance, offspring performance, and sow antioxidant status were analyzed for both studies using the mixed model procedure of SAS (v9.4, SAS Inst. Inc., Cary, NC) considering the effect of dietary supplementation, where sow was the experimental unit and sow(block) as the random effect for both suckling and postwean performance. In study 2, because of the different housing locations in the postweaning period, location was included in the model for postweaning performance. Because space allowance per pig in the nursery period (up to 42 days after weaning) was above requirements (31), it was not considered in the statistical analysis. In study 1, an interaction between dietary treatments was not detected; thus, the interaction term was removed, and treatment groups (CON, YC, MO, and YCMO) were considered independent. Weight category distribution and category change were analyzed using the Freq procedure in SAS (v9.4) and considered as main effects of base (control and CitriStim) and oil (carrier and mint oil). Correlation between milk/colostrum and sow serum circulating IGF-1 was assessed with the mixed model procedure considering the effect of dietary supplementation where sow was the experimental unit and sow (block) was the random effect. For the analysis of the suckling pig performance, a covariate of age was used to determine wean BW and ADG. Significant differences were reported at *p* < 0.05, and tendencies for significance were reported when 0.05 ≤ *p* ≤ 0.10 using the Tukey's mean separation test.

## Results

### Study 1

#### Farrowing and Suckling Performance

One sow in each of CON and YCMA and their litters were removed from the trial due to sow feed consumption issues in lactation and refusal to nurse piglets. Sow BW at lactation and weaning was not affected by maternal dietary treatment (Supplementary Table 1). Back fat at start of trial was lower (*p* < 0.05) in females assigned to YC compared to MO and YCMO females. A similar pattern was observed at weaning, where BF of YC-fed females tended to be lower (*p* < 0.10) than that of YCMO females. No difference was observed for birth weight and number of stillborn, born alive, mummies, and piglets weaned/litter (Table 3). Piglets from CON females were lightest (*p* < 0.05) at weaning due to lower daily gain (*p* < 0.05) compared to piglets from all other groups with no difference between YC, MO, and YCMO piglets.

#### Sow Antioxidant Activity

No effect of dietary treatments was noted for serum concentration of SOD at day 110 of gestation or at weaning (Table 3). There was no effect of treatment on SOD content in colostrum or milk, but a reduction in SOD as lactation progressed (i.e., colostrum vs. milk) was observed. Similarly, there was no impact of treatment on GSH content in colostrum but a tendency for greater (*p* < 0.10) GSH content in milk from YCMO sows.

#### Postwean Performance

Pigs fed YC_wean_ were heaviest (*p* < 0.05), and pigs fed YCMO_wean_ were lightest (*p* < 0.05) at d6 postweaning (Table 4). No difference in BW at d13, d19, and d35 was detected. Daily gain was greater (*p* < 0.05) in pigs fed CON_wean_ than YCMO_wean_ in the first 6 days postwean. Average daily feed intake was lowest (*p* < 0.05) for MO_wean_-fed pigs in phase 2 (d6–d13), greatest (*p* < 0.05) for groups fed the combination diet in phase 3 (d13–d19), and greatest (*p* < 0.05) for CON_wean_-fed pigs in phase 4 (d19–d35). Gain to feed was lowest (*p* < 0.05) in YCMO_wean_ and highest (*p* < 0.05) in MO_wean_-fed pigs in phase 2, lowest (*p* < 0.05) in YCMO_wean_-fed pigs in phase 3, and not different between treatments in phase 4. For the entire period, pigs fed the YCMO_wean_ diet had the lowest (*p* < 0.05) G:F ratio compared to CON_wean_ and MO_wean_.

The proportion of lightweight pigs at weaning deemed average and heavy by d35 after weaning increased regardless of dietary treatment (Table 5). At weaning, a greater proportion (*p* < 0.07) of MO_wean_-fed piglets were still deemed lightweight at d6 after weaning compared to CON_wean_ pigs. By d35 after weaning, 23% of MO_wean_ pigs had moved to average or heavyweight compared to 12% of lightweight CON_wean_ pigs. In pigs deemed heavy at weaning, a greater proportion (*p* < 0.05) receiving the YC_wean_ diets were assigned to the heavy category at d6 postweaning and likely reflects the heavier wean weight in this group (Table 4). A proportion of pigs deemed heavyweight at weaning (i.e., 5–7%) were observed to fall back and become average or lightweight in the postweaning period. However, at d35, 53% of YC_wean_-fed pigs deemed heavy at weaning were still heavy compared to 33% of CON_wean_-fed pigs.

**Table 5:** Distribution (%) of pigs that were deemed light and heavy at weaning throughout the postweaning period (study 1)^1^.

	**CON**	**YC**	**Chisq**	**CON**	**MO**	**Chisq**
**Lightweight**						
Day 6						
<6.06 kg	64.0	65.0	0.980	50.0	80.9	0.072
6.06–7.60 kg	32.0	30.0		45.8	14.3	
≥7.65 kg	5.0	4.0		4.2	4.8	
Day 13						
<8.00 kg	56.0	70.0	0.570	54.2	71.4	0.446
8.05–10.00 kg	25.0	40.0		41.6	23.8	
≥10.05 kg	4.0	5.0		4.2	4.8	
Day 19						
<9.96 kg	64.0	64.7	0.965	60.9	68.4	0.513
9.96–12.50 kg	28.0	29.4		34.8	21.1	
≥12.55 kg	8.0	5.9		4.3	10.5	
Day 35						
<17.05 kg	45.8	50.0	0.443	38.1	58.8	0.443
17.05–22.00 kg	37.5	35.7		42.9	29.4	
≥22.05 kg	16.7	14.3		19.0	11.8	
**Heavyweight**						
Day 6						
<6.06 kg	7.0	5.7	0.014	5.0	7.9	0.922
6.06–7.60 kg	56.2	30.2		47.5	39.2	
≥7.65 kg	36.8	64.1		47.5	52.9	
Day 13						
<8.00 kg	10.5	5.8	0.302	8.5	8.0	0.994
8.05–10.00 kg	42.1	32.7		37.3	38.0	
≥10.05 kg	47.4	61.5		54.2	54.0	
Day 19						
<9.96 kg	10.7	5.8	0.341	10.3	6.0	0.504
9.96–12.50 kg	42.9	34.6		34.5	44.0	
≥12.55 kg	46.4	59.6		55.2	50.0	
Day 35						
<17.05 kg	14.8	7.84	0.112	10.71	12.24	0.491
17.05–22.00 kg	51.85	39.22		41.07	51.02	
≥22.05 kg	33.33	52.94		48.21	36.73	

1 *Weaned pigs were provided the same dietary treatment that had been applied to their respective dams during lactation. In each phase, a whole yeast cell component (CitriStim, ADM Animal Nutrition, Quincy, IL) was added to the control (CON) diet at 0.2% at the expense of corn to create the YC_wean_ diets; peppermint oil was added at 0.01% to the control and YC_wean_ diets to create MO_wean_ and YCMO_wean_ diets. The C:18 fat base was included in the control and YC_wean_ diets at 0.01% to balance fat inclusion across all treatments. Interaction effect was not significant; therefore, only main effects are reported*.

### Study 2

#### Farrowing and Suckling Performance

There was no effect of maternal diet on sow BW or BF throughout the trial (Supplementary Table 1). Sows in the GT group tended to have greater (*p* < 0.10) feed intake in gestation than YC. There was no difference in sow lactation feed intake or litter characteristics at birth (Supplementary Table 1; Table 6). Pigs removed (died or fall back) were 4–7% lower in MO and GT litters. Piglet BW was not different at birth or weaning, and ADG was similar across treatment groups in the suckling period.

**Table 6:** Reproductive performance of sows fed one of four treatments throughout gestation and lactation, litter performance in the suckling phase, sow antioxidant status, and immunological profile of piglets and sow milk (study 2^1^)^4^^,^^5^.

**Items**	**CON**	**YC**	**MO**	**GT**	**SEM**	***p*-value**
Sow per treatment	13	14^2^	12	14		
Litter, average						
Born alive	15.0	13.8	14.8	14.4	0.93	0.855
Stillborn	1.00	1.00	1.60	1.10	0.54	0.794
Mummies	0.61	0.58	0.42	1.00	0.33	0.616
Weaned	12.8	12.2	12.5	12.4	0.59	0.927
% Removed	22.2	21.8	15.6	17.5		
Piglet BW, kg						
Birth^3^	1.34	1.34	1.35	1.36	0.03	0.952
d4 of lactation	1.76	1.85	1.83	1.83	0.04	0.253
d7 of lactation	2.39	2.39	2.43	2.41	0.06	0.945
Wean, suckling	5.55	5.66	5.69	5.90	0.14	0.313
Wean, all	5.41^x^^,^^y^	5.34^y^	5.63^x^^,^^y^	5.77^x^	0.14	0.040
kg weaned/sow, suckling	67.6	64.0	70.8	71.0	4.53	0.617
Piglet ADG, g/day						
days 0–4	80	90	90	100	1.00	0.711
days 4–7	150	230	110	140	5.00	0.274
Wean, all	200^b^	210^b^^,^^x^	220^ab^	230^a^^,^^y^	1.00	0.011
Wean, suckling	200	210	210	220	1.00	0.262
GSH, U/ml						
Colostrum	3.38	3.01	2.95	3.21	0.85	0.976
Milk, lactation day 4	7.16	7.43	7.38	7.61	1.72	0.998
Milk, lactation day 14	7.92	11.8	12.5	9.77	2.29	0.464
SOD serum, U/ml						
Breeding	9.94	9.83	9.79	9.72	1.34	0.999
D110 of gestation	18.5	18.7	13.5	16.6	2.75	0.360
Weaning	9.76	11.82	10.69	10.09	1.38	0.504
IGF-1, ng/ml						
Serum, d110 of gestation	52.9	56.8	58.0	59.9	4.60	0.624
Colostrum	864.5	876.0	882.6	934.6	46.60	0.723
Milk, lactation day 4	298.8	306.2	303.3	299.4	3.00	0.078
Milk, lactation day 14	298.2	302.6	301.8	298.2	4.17	0.666
Colostrum immunocrit	0.37	0.40	0.36	0.39	0.04	0.896
Piglet immunocrit	0.17	0.17	0.17	0.19	0.02	0.288
Piglet immunocrit based on weight distribution						**Chi-sq**
Light (<1.1 kg)	0.18	0.14	0.16	0.17	0.03	0.775
Average (1.15–1.65 kg)	0.15	0.18	0.16	0.19		
Heavy (>1.65 kg)	0.17	0.17	0.18	0.21		

1 *The whole yeast cell, mint oil, and γ-tocopherol were top dressed at 50 g/day and supplied 3.1 g whole yeast cell/day, 10 ppm active peppermint oil/day, and 200 ppm γ-tocopherol/day, respectively*.

2 *One sow from the YC treatment group was euthanized during farrowing due to a twisted uterus*.

3 *Piglets were weighed within 24 h of parturition*.

4 *Values denoted with superscript*

x,y *indicate a tendency (p < 0.10)*.

5 *Values denoted with superscript*

a,b,c,d *indicate a significance (p < 0.05)*.

When BW at weaning for all offspring (those reared on the dam and those artificially reared) was assessed, GT offspring tended to weigh more than YC offspring (5.77 and 5.34 kg, 0.14 kg SEM, respectively; Table 6). Offspring from GT-fed sows also gained more than CON offspring and tended to gain more than YC offspring (0.23 vs. 0.20 and 0.21 kg/day, 0.01 kg/day SEM, respectively). Growth performance of offspring that were reared artificially was monitored in the suckling phase only.

#### Sow Antioxidant Activity

Colostrum and milk GSH content were not affected by maternal treatment with a 4% greater concentration in milk than colostrum (Table 6). SOD content in sow serum was not affected by maternal treatment.

#### Markers of Piglet Robustness

There was a tendency for an effect of maternal treatment on serum concentration of IGF-1 and d4 milk, but no differences between treatments were noted based on Tukey's adjustment (Table 6). IGF-1 in colostrum was almost 2-fold higher than in milk. Colostrum immunocrit was not affected by maternal dietary treatment. There was no difference in d2 immunocrit value (Table 6) by sow treatment or birth weight category.

#### Postwean Growth Performance

Pigs from YC sows were heavier (*p* < 0.05) at d29 after weaning than pigs from CON sows (Table 7), which is in part due to greater (*p* < 0.05) feed intake and daily gain. Similarly, pigs from MO and GT sows tended to be heavier (*p* < 0.10) at d29 after weaning than pigs from CON sows. An increasing proportion of pigs from CON-fed sows fell back into the lightweight category by d29 after weaning (Table 7).

**Table 7:** Performance of weaned pigs from d0 to 29 after weaning from sows provided feed additives in gestation and lactation (Study 2)^1^^,^^3^^,^^4^.

**Items**	**CON**	**YC**	**MO**	**GT**	**SEM**	***p-*value**
Body weight, kg						
d4	5.30	5.50	5.39	5.27	0.36	0.916
d14	7.51	8.10	7.89	8.19	0.32	0.176
d29	12.37^b^^,^^y^	13.83^a^^,^^x^	13.85^x^	13.65^x^	0.54	0.031
Daily gain, kg/day						
d0–4	0.04	0.06	0.01	0.05	0.02	0.118
d4–14	0.21^b^^,^^y^	0.24^a^^,^^x^	0.24^x^	0.24^a^^,^^x^	0.01	0.013
d14–29	0.33^y^	0.39^x^^,^^y^	0.41^x^	0.37^x^^,^^y^	0.02	0.050
d0–29	0.23	0.24	0.24	0.24	0.02	0.948
Daily feed intake, kg/day						
d4–14	0.21^b^^,^^y^	0.26^a^	0.18^b^	0.25^a^^,^^x^	0.02	0.002
d14–29	0.42^b^^,^^y^	0.56^a^^,^^x^	0.45^x^^,^^y^	0.54^x^	0.05	0.019
d0–29	0.28	0.36	0.29	0.34	0.03	0.050
Gain: feed						
d4–14	0.90^b^^,^^y^	0.93^a^	1.23^b^	0.94^a^^,^^x^	0.07	0.004
d14–29	0.85	0.72	0.94	0.79	0.09	0.227
d0–29	0.88	0.69	0.82	0.69	0.13	0.279
Distribution of piglets^2^						**Chi-sq**
Day 4						
<5.1 kg	29.1	27.6	25.5	19.7		0.188
5.15–7.20 kg	50.0	44.8	55.0	51.3		
≥7.26 kg	21.0	27.6	19.5	28.9		
Day 14						
<7.26 kg	34.5	27.9	20.0	22.2		0.114
7.27–9.90 kg	45.3	48.1	55.9	51.0		
≥9.95 kg	20.3	24.0	24.1	26.9		
Day 29						
<12.60 kg	36.5	25.5	17.4	23.5		0.013
12.65–16.50	41.9	46.4	56.3	53.9		
≥16.55	21.6	28.1	26.4	23.5		

1 *Feed additives were added to the control (CON) diet in gestation and lactation. The whole yeast cell, mint oil, and γ-tocopherol were top dressed at 50 g/day and supplied 3.1 g whole yeast cell/day, 10 ppm active peppermint oil/day, and 200 ppm γ-tocopherol/day, in YC, MO, and GT, respectively. Piglets were weaned at 20 ± 2 days of age to common diets in a three-phase nursery feeding program (phase 1, d0–d4; phase 2, d4–d14; phase 3, d14–d29)*.

2 *Weight distribution categories based on average and standard deviation of all pigs at each weigh period. Categories represent <1 SD of the average, average ± 1SD, and >1 SD of the average*.

3 *Values denoted with superscript*

x,y *indicate a tendency (p < 0.10)*.

4 *Values denoted with superscript*

a,b,c,d *indicate a significance (p < 0.05)*.

At the on-site facility, lightweight pigs from MO and GT sows were heavier (*p* < 0.05) at d42 and tended to be heavier (*p* < 0.10) up to d98 (Table 8). No difference in BW was observed for heavyweight pigs throughout the finishing period; although, pigs from MO-fed sows were around 3 kg heavier than all other treatment groups at d126. Lightweights from the MO-fed sow group had higher gain (*p* < 0.05) than all other treatments throughout the finishing period, while pigs from GT sows tended to have lesser gain. Heavyweight pigs from CON sows tended to gain less (*p* < 0.10) than the MO or GT sows from d98 up to d126.

**Table 8:** Grow-finish (d29–d126 after weaning) performance of offspring deemed light and heavy at weaning from sows provided feed additives during gestation and lactation and weaned to the on-site facility (study 2)^1^^,^^4^^,^^5^.

**Items**	**CON**	**YC**	**MO**	**GT**	**SEM**	***p*-value**
Lightweight^2^ pigs						
Body weight, kg						
d42	16.1^c^	17.8^bc^	19.3^ba^	20.5^a^	0.86	<0.0001
d70	34.1^x^	36.1^x^^,^^y^	38.8^y^	38.6^z^	1.72	0.034
d98	55.8^x^	58.1^x^^,^^y^	62.3^y^	60.2^x^^,^^y^	2.54	0.096
d126	85.0	84.2	92.6	87.6	3.71	0.178
Daily gain, kg/day						
d29–d42	0.37^b^	0.32^b^^,^^x^	0.57^a^	0.17^c^^,^^y^	0.05	<0.0001
d42–d70	0.47^b^	0.39^b^^,^^x^	0.75^a^	0.20^c^^,^^y^	0.07	<0.0001
d70–d98	0.49^b^^,^^y^	0.42^b^	0.77^a^^,^^x^	0.28^b^	0.08	0.002
d98–d126	0.67^b^	0.52^bc^	1.01^a^	0.33^c^	0.11	0.0001
Heavyweight^3^ pigs						
Body weight, kg						
d42	26.0	28.2	27.3	26.1	1.10	0.132
d70	49.0	50.6	50.0	48.8	1.64	0.596
d98	73.4	74.2	74.8	72.9	2.46	0.870
d126	102.5	102.4	105.0	102.2	3.24	0.850
Daily gain, kg/day						
d29–d42	0.35	0.67	0.56	0.62	0.21	0.109
d42–d70	0.44	0.61	0.65	0.70	0.19	0.163
d70–d98	0.43^y^	0.69^x^^,^^y^	0.68^x^^,^^y^	0.81^x^	0.23	0.078
d98–d126	0.52^y^	0.79^x^^,^^y^	0.82^x^	0.90^x^^,^^y^	0.23	0.077

1 *Feed additives were added to the control (CON) diet in gestation and lactation. The whole yeast cell, mint oil, and γ-tocopherol were top dressed at 50 g/day and supplied 3.1 g whole yeast cell/day, 10 ppm active peppermint oil/day, and 200 ppm γ-tocopherol/day, in YC, MO, and GT, respectively*.

*Pigs were provided common diets in a six-phase nursery feeding program of ~21 days/phase*.

2 *Lightweight pigs represent pigs weighing 5.13 kg or less at weaning*.

3 *Heavyweight pigs represent pigs weighing 7.05 kg or more at weaning*.

4 *Values denoted with superscript*

x,y *indicate a tendency (p < 0.10)*.

5 *Values denoted with superscript*

a,b,c,d *indicate a significance (p < 0.05)*.

At the off-site facility, lightweight pigs from YC sows were lightest at d42 and tended to remain lighter through d126 (Table 9). There was no detectable difference in BW of heavyweight pigs by maternal treatment; however, pigs from YC, MO, and GT sows were ~3, 8, and 6 kg, respectively, heavier at d126 than pigs from CON sows. These differences in BW are in part reflective of differences in daily gain (*p* < 0.05).

**Table 9:** Grow-finish (d29–d126 after weaning) performance of offspring deemed light and heavy at weaning from sows provided feed additives during gestation and lactation and weaned to an off-site facility^1^^,^^4^.

**Items**	**CON**	**YC**	**MO**	**GT**	**SEM**	***p*-value**
**Lightweight^2^ pigs**						
Body weight, kg						
d42	18.3^x^^.^^y^	15.1^y^	19.1^x^	18.8^x^	1.60	0.027
d70	44.6	43.2	45.4	46.0	1.80	0.537
d98	74.4	70.0	71.4	74.6	2.30	0.238
d126	104.6	100.3	101.1	106.5	2.70	0.177
Daily gain, kg/day						
d29–d42	0.14	0.12	0.09	0.18	0.04	0.220
d42–d70	1.00	1.03	0.97	1.01	0.05	0.573
d70–d98	1.04^a^	0.94^c^	0.91^b^	1.02^ac^	0.03	0.006
d98–d126	1.03	1.04	1.02	1.02	0.06	0.979
**Heavyweight^3^ pigs**						
Body weight, kg						
d42	28.8	28.4	29.3	26.2	1.84	0.532
d70	58.3	61.5	61.4	58.4	3.03	0.532
d98	87.2	89.9	90.5	89.3	3.92	0.807
d126	112.1	115.7	120.4	118.7	4.64	0.276
Daily gain, kg/day						
d29–d42	0.26^a^	0.12^b^	0.18^ab^	0.18^ab^	0.03	0.038
d42–d70	1.04^x^	1.20^y^	1.16^x^^,^^y^	1.17^x^^,^^y^	0.06	0.054
d70–d98	0.92	0.98	1.01	1.07	0.05	0.128
d98–d126	0.95	1.01	1.09	1.11	0.06	0.181

1 *Feed additives were added to the control (CON) diet in gestation and lactation. The whole yeast cell, mint oil, and γ-tocopherol were top dressed at 50 g/day and supplied 3.1 g whole yeast cell/day, 10 ppm active peppermint oil/day, and 200 ppm γ-tocopherol/day, in YC, MO, and GT, respectively. Pigs were provided common diets in a six-phase feeding program of ~21 days/phase*.

2 *Lightweight pigs represent 5.13 kg or less at weaning*.

3 *Heavyweight pigs represent pigs weighing 7.05 kg or more at weaning*.

4 *Values denoted with superscript*

x,y *indicate a tendency (p < 0.10)*.

## Discussion

The objective of the study was to assess the impact of whole yeast cell, peppermint oil, and ɤ-tocopherol supplementation in sow diets and offspring diets in the nursery phase on sow antioxidant status and offspring performance in study 1, and the potential carryover effects of sow diet supplementation on offspring performance in the suckling and postweaning periods as in study 2. In relation to the sow, there were minor effects of feed additive inclusion on sow performance, including weight changes in gestation and lactation, and litter characteristics at birth. Potential attributes to the lack of effect on sow reproductive performance may be due to the number of pregnant females utilized. At least 100 sows/treatment is often stated as the minimum number of reproductive females needed to detect significant differences in reproductive performance due to treatment based on papers by Aaron and Hays (32, 33). While the total number of females used in these trials may limit conclusions for reproductive performance, the focus of these studies was offspring growth, for which replication was sufficient to detect meaningful differences (34). Another potential reason for the lack of difference on sow performance could be the difference in additive delivery in study 1, where whole yeast cell was mixed in the diet and the mint oil was added as a top dress. There is greater potential for loss of additive or inconsistent daily intake of additive due to sow feeding behavior when included as a top dress. As well, a constant daily top dress does not account for differences in sow daily feed intake. In study 2, to balance the risk across treatments, all test additives were top dressed. However, throughout both studies, feeder management was monitored very closely to ensure limited residual feed following each feeding period. In addition, the lack of difference in daily feed intake, particularly in lactation, suggests that the impact of differences in additive delivery on sow performance was likely minor. Minor impacts on sow performance with the addition of phytochemicals or yeast cell products have been reported previously (14, 15, 19). Similarly, there were minor alterations in sow antioxidant status; however, there were clear benefits in relation to offspring performance in the suckling and/or postweaning period with supplementation of the sow diet.

As both whole yeast cell and mint oil are reported to have different modes of action in relation to animal performance, a combination treatment was included in lactation and nursery diets for study 1 to determine if the inclusion of both would provide more benefits to both dam and offspring than if fed separately. However, the inclusion of both whole yeast cell and mint oil in study 1 did not offer an additive effect on the performance of lactating sows or offspring during the suckling and early postwean period. Whole yeast cell product used in this work is derived from *P. guilliermondii*, which possesses antimicrobial activity allowing for the binding of pathogens in the small intestine and has been reported to modulate immunomodulatory effects in broilers (35). Peppermint oil, or *Mentha x piperita*, is a part of the Mentha family, which is known to possess antioxidant activity. However, this species of Mentha was reported to induce apoptotic cell death in *Saccharomyces cerevisiae* (36). While it is unknown if mint oil has a similar effect on *P. guilliermondii*, the mint oil used in this work may have negated the antimicrobial benefits of the yeast strain. The potential for mint oil to induce apoptotic cell death in the yeast cells may explain the lack of additive effect in YCMO-fed sows and piglets. Performance of offspring from YCMO-fed sows was similar to that of offspring from MO-fed sows. If the mint oil reduced activity of the whole yeast cell, animal performance would be expected to be the same as females supplemented with mint oil only.

Selection for a more prolific sow has resulted in increased number born alive per litter, greater within-litter variation at birth, and increased proportion of lightweight pigs (37). Concern regarding the performance of small pigs in the postweaning period results in increased labor and feed costs. An increase in feed and management costs was reported for lighter pigs, due to the longer time and increased feed required for them to reach the required minimum commercial slaughter weight (38). Variation at weaning and up to slaughter can negatively impact the monetary gain for producers as lightweight animals need more days to market than their heavier counterpart, which results in increased feed cost. In the current work, inclusion of mint oil or ɤ-tocopherol provided assistance to lightweight animals during the suckling period, in particular in study 2 when fallback pigs (artificially reared pigs) were included, which suggests long-lasting effects from *in utero* exposure allowed these fallbacks to thrive even if not suckling from the dam. This benefit was also evident during the postweaning period in study 2 in relation to the percentage of animals that remained in the lightweight category and bodyweight. This work suggests that inclusion of mint oil in sow diets conveys a particular benefit to “opportunity” pigs; those that are light or borderline average weight could potentially gain weight to become average and in turn reduce days to market. It has been reported that heavier birth weight piglets consume about 30% more milk than their lighter littermates (39) and are commonly expected to experience fewer negative impacts of weaning. However, results from study 1 suggest there is risk of heavy pigs losing their heavyweight status and that inclusion of whole yeast cell provided a means for pigs deemed heavyweight at birth and weaning to maintain their heavyweight status in the postweaning period. The specific mechanism for this benefit is unclear. The lack of a similar response in study 2 may be related to supplementation times, where whole yeast cell was supplemented in both the sow diet and the postweaning diets in study 1 and in the sow diet only in study 2. Within study 2, the difference in BW between pigs at the on-site and off-site facility at an equivalent day postweaning suggests an environmental effect; however, because pigs at each facility were from different farrowing groups and weaned to the respective facilities on different calendar days (i.e., 28 days between weaning of block 1 and 2 in study 2), direct comparisons were not made.

Differences in offspring response to whole yeast cell and mint oil in the suckling and postweaning period between study 1 and study 2 can aid in determining the time of maternal supplementation to achieve the greatest offspring benefit. Maternal diet supplementation of whole yeast cell and mint oil in lactation only improved suckling offspring performance based on BW at weaning and kilogram of pig weaned/sow, respectively, with limited effect after weaning. An effect on wean weight was not observed in other similar phytochemical supplementation studies (14–16). However, slightly lower supplementation of whole yeast cell through both gestation and lactation provided benefit to offspring performance that was maintained even after weaning. Based on these studies, maternal diet supplementation in both gestation and lactation had longer-term benefit to offspring growth. Inclusion of peppermint oil would assist reducing ROS concentration in lipid membranes, potentially reducing gut permeability. Coupled with the inclusion of CitriStim, which has antimicrobial activity and could bind to pathogens found in the intestinal lumen, the idea that the inclusion of either one or even the combination of the two would aid in the prevention of poor growth in the early postwean period. However, as observed in the lactation period, combination of the additives diet did not result in a better performance compared to pigs that were given the feed additives separately.

The antioxidant defense system is necessary in reducing free ROS found in the body due to oxidative stress. The periods of gestation and lactation are considerably stressful time for the dam and are found to result in an increased oxidative status in the female. With newly weaned animals, the occurrence of oxidative stress has been noted to result in reduced growth performance, increased disease incidence, and even death (13). These outcomes are related to the denaturing of tight junction proteins lining the small intestine as a result of the overproduction of free radicals. With reduced barrier function of the intestinal epithelium as a result of disrupted tight junction proteins, the potential for undigested feedstuff and toxins in the intestinal lumen to pass the basal lateral membrane increases. Measuring the concentration of certain antioxidants that play a vital role in the defense system would give insight to the efficiency of the antioxidant defense system. To that end, based on the assays used in this work, a higher SOD value represents a greater need of the SOD enzyme for dismutation of superoxide radical and greater free radicals, insinuating a higher state of stress. Conversely, a lower GSH value insinuates greater oxidative state. SOD concentration at d110 of gestation in study 1 was higher in YC- and MO-fed sows, potentially indicating that the females were at a greater level of oxidative stress at the start of the study. At weaning, the serum concentration of SOD for all treatment groups was similar in value, which may provide evidence of a greater reduction in oxidative stress in the YC and MO sows and may, in part, explain differences in offspring growth. In both studies, there appeared to be an effect of time on sow serum SOD content where concentration was close to 2-fold greater at d110 of gestation relative to breeding and weaning. This observation suggests that late gestation is a period of greater oxidative stress than lactation and is supported by the lesser GSH concentration in d4 milk compared to colostrum. A similar reduction in levels of oxidative markers in milk as lactation progresses was reported when resveratrol was supplemented in sow gestation and lactation diets (15). Thus, sow diet alterations to address oxidative status may be most effective during gestation, particularly late gestation, and are supported by the improved performance of YC and MO offspring in the postweaning period in study 2 and opportunity pigs in both studies. While not measured in this work, it would be advantageous in future studies to measure serum concentration of antioxidants following weaning in offspring to determine the effects on offspring antioxidant defense system.

Measuring antioxidant concentration in colostrum and milk provides insight into the oxidative status of the dam during different periods of lactation, as milk is noted to be a carrier of bioactive compounds such as antioxidants (40). Therefore, the concentration of antioxidants in the body, be it depleted due to an onslaught of reactive oxygen species or at baseline when the animal is not stressed, is well-represented in the milk. The use of this biological sample type provides a minimally invasive means of assessing sow oxidative status. Analysis of milk also can be utilized to determine the concentration of antioxidants that are potentially being transferred to the offspring. In humans, antioxidant transfer is reported to occur *in utero via* the placenta (41); however, that is not the sole point of antioxidant uptake that can occur in the offspring's life. In study 1, supplementation of test additives occurred during the suckling period. The finding that lightweight offspring from the MO group became average weight while heavyweight offspring from the YC group were able to maintain their heavyweight status at weaning and at the end of the nursery period compared to CON indicates exposure to antioxidant compounds *via* colostrum and milk could enhance the offspring's own antioxidant defense system. This potentially better prepares the offspring's defense against large influxes of reactive oxygen species that can arise from the imbalance between prooxidants and antioxidants (9), which can arise during periods of stress. The MO group at both off- and on-site facilities weighing heaviest at the end of study 2 supports the speculation that longer-term maternal supplementation similarly benefits offspring ability to withstand periods of stress.

Gilts and sows past their fifth parity are found to possess a lower antioxidant capacity (42). Lower capacity may indicate that these females are less successful in reducing free radical concentration compared to their second to fourth parity counterparts. Thus, they may be likely to experience oxidative stress more frequently or undergo it at a faster rate. The concentration of SOD appeared to be higher in gilts during lactation, which may indicate that intervention of oxidative stress in the sow may only need to occur in specific. Similarly, it has been suggested that it may be best to supplement sows before their first ever farrowing and those that have had five litters or more (16, 42).

One of the vital components of colostrum is immunoglobins, more specifically immunoglobin G (IgG), and other immune components that can directly or indirectly influence the immune system of the piglet (43). Because of the immature immune system of piglets at birth (43), intake of immunoglobins is essential for the offspring to obtain passive immunity from the dam's immune components. Through the measurement of immunocrit in both piglet sera and colostrum, one can evaluate the concentration of immunoglobins found in the dam's milk as well as determine suckling ability of the offspring. As the immunocrit values at d2 did not differ by weight category or treatment, it suggests that differences in piglet weight at weaning may be more related to *in utero* fetal development and/or milk nutrient supply than alteration of the immunological capacity at birth.

IGF-1 is one of several immune factors that are contained in colostrum. IGF-1 is thought to be involved in growth and development of young animals (44). Serum concentration of the growth hormone in offspring is utilized as an indicator of nutritional status (45, 46). No effect of maternal dietary treatment was noted for IGF-1 in d110 serum, colostrum, and d4 and d14 milk samples. A pattern was denoted that as the lactation period progressed, the concentration of IGF-1 decreased within milk. It was found that the concentration of roaming IGF-1 in colostrum for swine is 4 to 17 nM, with mature milk ranging from 1 to 3 nM (47). The concentration of IGF-1 in colostrum and milk determined in the study is much higher than the values found in the literature. The lack of difference in sow sera, colostrum, and milk IGF-1 suggests some other factor are involved in the improvement of piglet performance observed in both studies.

## Conclusion

Based on these studies, the end of gestation prior to farrowing is the period of greatest oxidative stress. Dietary inclusion of whole yeast cell, peppermint oil, and ɤ-tocopherol in sow diets improved offspring performance during both the suckling and postweaning periods. In particular, whole yeast cell and ɤ-tocopherol supplementation in sow diets during lactation resulted in heavier offspring, while prenatal and postnatal exposure to mint oil benefited lightweight pig up to market. Overall, this work suggests that the inclusion of whole yeast cell or peppermint oil in sow and weaned pig diets could aid producers in reducing costs, labor, and time needed to support fallback or lightweight animals.

## Data Availability Statement

The original contributions presented in the study are included in the article/Supplementary Material, further inquiries can be directed to the corresponding author/s.

## Ethics Statement

The animal study was reviewed and approved by Institutional Animal Care and Use Committee at South Dakota State University (SDSU; IACUC #17-072A).

## Author Contributions

LH: data curation, formal analysis, investigation, and writing–original draft preparation. JD and JW: conceptualization and writing–reviewing and editing. AH: writing–reviewing and editing. CL: supervision, project administration, and funding acquisition. All authors reviewed this manuscript.

## Conflict of Interest

JD, JW, and AH were employed by the company ADM Animal Nutrition. The remaining authors declare that the research was conducted in the absence of any commercial or financial relationships that could be construed as a potential conflict of interest.

## References

[1] Kim SW Weaver AC Shen YB Zhao Y. Improving efficiency of sow productivity: nutrition and health. J Anim Sci Biotechnol. (2013) 4:26. 10.1186/2049-1891-4-26 23885840 PMC3733949

[2] Foxcroft GR Dixon WT Novak S Putman CT Town SC Vinsky MDA. The biological basis for prenatal programming of postnatal performance in pigs. J Anim Sci. (2006) 84:E105–12. 10.2527/2006.8413_supplE105x 16582081

[3] Quesnel H Brossard L Valancogne A Quiniou N. Influence of some sow characteristics on within-litter variation of piglet birth weight. Animal. (2008) 2:1842–9. 10.1017/S175173110800308X 22444091

[4] Kitt SJ. How to drive efficiencies in pork production: what are the gaps in knowledge and obstacles? J Anim Sci. (2020) 98(Suppl. 3):24–5. 10.1093/jas/skaa054.043

[5] Yang YX Heo S Jin Z Yun JH Choi JY Yoon SY. Effects of lysine intake during late gestation and lactation on blood metabolites, hormones, milk composition and reproductive performance in primiparous and multiparous sows. Anim Reprod Sci. (2009) 112:199–214. 10.1016/j.anireprosci.2008.04.031 18547756

[6] McPherson RL Ji F Wu G Blanton JR Kim SW. Growth and compositional changes of fetal tissues in pigs. J Anim Sci. (2004) 82:2534–40. 10.2527/2004.8292534x 15446468

[7] Koketsu Y Dial GD Pettigrew JE King VL. Influence of feed intake during individual weeks of lactation on reproductive performance of sows on commercial farms. Livest Prod Sci. (1997) 49:217–25. 10.1016/S0301-6226(97)00050-X

[8] Vadmand CN Krogh U Hansen CF Theil PK. Impact of sow and litter characteristics on Colostrum yield, time for onset of lactation, and milk yield of sows. J Anim Sci. (2015) 93:2488–500. 10.2527/jas.2014-8659 26020344

[9] Ighodaro OM Akinloye OA. First line defence antioxidants-superoxide dismutase (SOD), catalase (CAT) and glutathione peroxidase (GPX): their fundamental role in the entire antioxidant defence grid. Alexandria J. Med. (2018) 54:287–93. 10.1016/j.ajme.2017.09.001

[10] Fellenberg MA Speisky H. Antioxidants: their effects on broiler oxidative stress its meat oxidative stability. World's Poult Sci J. (2007) 62:53–70. 10.1079/WPS200584

[11] Liang F Jiang S Mo Y Zhou G Yang L. Consumption of oxidized soybean oil increased intestinal oxidative stress and affected intestinal immune variables in yellow-feathered broilers. Asian Austral J Anim Sci. (2015) 28:1194–201. 10.5713/ajas.14.0924 26104529 PMC4478489

[12] Shurson GC Kerr BJ Hanson AR. Evaluating the quality of feed fats and oils and their effects on pig growth performance. J Anim Sci Biotechnol. (2015) 6:10. 10.1186/s40104-015-0005-4 25844168 PMC4384276

[13] Zheng P Yu B He J Tian G Luo Y Mao X. Protective effects of dietary arginine supplementation against oxidative stress in weaned piglets. Br J Nutr. (2013) 109:2253–60. 10.1017/S0007114512004321 23176743

[14] Tan C Wei H Sun H Ao J Long G Jiang S. Effects of dietary supplementation of oregano essential oil to sows on oxidative stress status, lactation feed intake of sows, piglet performance. Biomed Res Int. (2015) 2015:525218. 10.1155/2015/525218 26539506 PMC4619846

[15] Meng Q Guo T Li G Sun S He S Cheng B. Dietary resveratrol improves antioxidant status of sows and piglets and regulates antioxidant gene expression in placenta by Keap1-Nrf2 pathway and Sirt1. J Anim Sci Biotechnol. (2018) 9:34. 10.1186/s40104-018-0248-y 29713468 PMC5909222

[16] Lipiński K Antoszkiewicz Z Mazur-Kuśnirek M Korniewicz D Kotlarczyk S. The effect of polyphenols on the performance and antioxidant status of sows and piglets. Ital. J Anim Sci. (2019) 18:174–81. 10.1080/1828051X.2018.1503043

[17] Reyes-Camacho D Vinyeta E Pérez JF Aumiller T Criado L Palade LM. Phytogenic actives supplemented in hyperprolific sows: effects on maternal transfer of phytogenic compounds, colostrum and milk features, performance and antioxidant status of sows and their offspring, and piglet intestinal gene expression. J Anim Sci. (2020) 98:1–13. 10.1093/jas/skz390 31910258 PMC6981091

[18] Riachi LG De Maria CAB. Peppermint antioxidants revisited. Food Chem. (2015) 176:72–81. 10.1016/j.foodchem.2014.12.028 25624208

[19] Bass BE Tsai TC Yang H Perez V Holzgraefe D Chewning J. Influence of a whole yeast product (Pichia guilliermondii) fed throughout gestation and lactation on performance and immune parameters of the sow and litter. J Anim Sci. (2019) 97:1671–8. 10.1093/jas/skz060 30770711 PMC6447256

[20] Niki E Traber MG. A history of vitamin E. Ann Nutr Metab. (2012) 61:207–12. 10.1159/000343106 23183290

[21] Takahashi K Tatsuya K Takeda S Takeda M Koshia R Nakayama M. γ-Tocopherol, but not α-tocopherol, potently inhibits neointimal formation induced by vascular injury in insulin resistant rats. J Mole Cell Cardiol. (2006) 41:544–54. 10.1016/j.yjmcc.2006.06.010 16876819

[22] Jiang Q Lykkesfeldt J Shigenaga MK Shigeno ET Christen S Ames BN. γ-Tocopherol supplementation inhibits protein nitration and ascorbate oxidative in rats with inflammation. Free Radic Bio Med. (2002) 33:1534–42. 10.1016/S0891-5849(02)01091-2 12446211

[23] Dey P Mah E Li J Jalili T Symons D Bruno RS. Improved hepatic γ-tocopherol status limits oxidative and inflammatory stress-mediated liver injury in db/db mice with non-alcoholic steatohepatitis. J Func Foods. (2018) 40:670–8. 10.1016/j.jff.2017.12.007

[24] Chung MY Yeung SF Park HJ Volek JS Bruno RS. Dietary α- and γ-tocopherol supplementation attenuates lipopolysaccharide-induced oxidative stress and inflammatory-related responses in an obese mouse model of non-alcoholic steatohepatitis. J Nutr Biochem. (2010) 21:1200–6. 10.1016/j.jnutbio.2009.10.006 20138495

[25] Devaraj S Leonard S Traber MG Jialal I. Gamma-tocopherol supplementation alone and in combination with alpha-tocopherol alters biomarkers of oxidative stress and inflammation in subjects with metabolic syndrome. Free Rad Bio Med. (2008) 44:1203–8. 10.1016/j.freeradbiomed.2007.12.018 18191645 PMC2676174

[26] Feldpausch JA Jourquin J Bergstrom JR Bargen JL Bokenkroger CD Davis DL. Birth weight threshold for identifying piglets at risk for preweaning mortality. Transl Ani Sci. (2019) 3:633–40. 10.1093/tas/txz076 32704833 PMC7200817

[27] Vallet JL Miles JR. The effect of farrowing induction on colostrum and piglet serum immunocrits is dependent on parity1. J Anim Sci. (2017) 95:688–96. 10.2527/jas.2016.0993 28380602

[28] Vallet JL Miles JR Rempel LA. A simple novel measure of passive transfer of maternal immunoglobulin is predictive of preweaning mortality in piglets. Vet J. (2013) 195:91–7. 10.1016/j.tvjl.2012.06.009 22831993

[29] Echternkamp SE Spicer LJ Gregory KE Canning SF Hammond JM. Concentrations of insulin-like growth factor-i in blood and ovarian follicular fluid of cattle selected for twins. Bio Repro. (1990) 43:8–14. 10.1095/biolreprod43.1.8 2393694

[30] Funston RN Moss GE Roberts AJ. Insulin-like growth factor-I (IGF-I) and IGF-binding proteins in bovine sera and pituitaries at different stages of the estrous cycle. Endocrinology. (1995) 136:62–8. 10.1210/endo.136.1.7530196 7530196

[31] Wolter BF Ellis M Curtis SE Parr EN Webel DM. Group size and floor-space allowance can affect weanling-pig performance. J Anim Sci. (2000) 78:2062–7. 10.2527/2000.7882062x 10947088

[32] Aaron DK Hays VW. Statistical techniques for the design and analysis of swine nutrition experiments. In: Lewis AJ Southern LL editors. 2nd ed. Swine Nutrition. Boca Raton: CRC Press (2001). p. 881–901.

[33] Aaron DK Hays VW. How many pigs? Statistical power considerations in swine nutrition experiments. J Anim. Sci. (2004) 82:E245–54. 15471805 10.2527/2004.8213_supplE245x

[34] Berndtson WE. A simple rapid and reliable method for selecting or assessing the number of replicates for animal experiments. J Anim Sci. (1991) 69:67–76. 10.2527/1991.69167x 2005039

[35] Shanmugasundaram R Selvaraj RK. Effect of killed whole yeast cell prebiotic supplementation on broiler performance and intestinal immune cell parameters. Poult Sci. (2012) 91:107–11. 10.3382/ps.2011-01732 22184435

[36] Ferreira P Cardoso T Ferreira F Fernandes-Ferreira M Piper P Sousa MJ. Mentha piperita essential oil induces apoptosis in yeast associated with both cytosolic and mitochondrial ROS-mediated damage. FEMS Yeast Res. (2014) 14:1006–14. 10.1111/1567-1364.12189 25065265

[37] Quiniou N Dagorn J Gaudré D. Variation of piglets' birth weight and consequences on subsequent performance. Livestock Prod Sci. (2002) 78:63–70. 10.1016/S0301-6226(02)00181-1

[38] Yuan TL Zhu YH Shi M Li TT Li N Wu GY. Within-litter variation in birth weight: impact of nutritional status in the sow. J Zhejiang Univ Sci B. (2015) 16:417–35. 10.1631/jzus.B1500010 26055904 PMC4471594

[39] Pluske JR Williams IH. Split weaning increases the growth of light piglets during lactation. Aust J Agric Res. (1996) 47:513–23. 10.1071/AR9960513

[40] Khan IT Nadeem M Imran M Ullah R Ajmal M Jaspal MH. Antioxidant properties of milk and dairy products: a comprehensive review of the current knowledge. Lipids Health Dis. (2019) 18:1–13. 10.1186/s12944-019-0969-8 30717735 PMC6362592

[41] Jauniaux E Cindrova-Davies T Johns J Dunster C Hempstock J Kelly FJ. Distribution and transfer pathways of antioxidant molecules inside the first trimester human gestational sac. J Clin Endocrinol Metab. (2004) 89:1452–8. 10.1210/jc.2003-031332 15001647

[42] Lipko-Przybylska J Kankofer M. Antioxidant defence of colostrum milk in consecutive lactations in sows. Ir Vet J. (2012) 65:4. 10.1186/2046-0481-65-4 22429994 PMC3338395

[43] Le Dividich J Rooke JA Herpin P. Nutritional and immunological importance of colostrum for the new-born pig. J Agric Sci. (2005) 143:469–85. 10.1017/S0021859605005642

[44] Laron Z. Insulin-like growth factor 1 (IGF-1): a growth hormone. Mol Pathol. (2001) 54:311–6. 10.1136/mp.54.5.311 11577173 PMC1187088

[45] Richards MW Wettemann RP Spicer LJ Morgan GL. Nutritional anestrus in beef cows: effects of body condition and ovariectomy on serum luteinizing hormone and insulin-like growth factor-I1. Biol Reprod. (1991) 44:961–6. 10.1095/biolreprod44.6.961 1873396

[46] Bossis I Wettemann RP Welty SD Vizcarra JA Spicer LJ Diskin MG. Nutritionally induced anovulation in beef heifers: ovarian and endocrine function preceding cessation of ovulation. J Anim Sci. (1999) 77:1536–46. 10.2527/1999.7761536x 10375231

[47] Donovan SM McNeil LK Jiménez-Flores R Odle J. Insulin-like growth factors and insulin-like growth factor binding proteins in porcine serum and milk throughout lactation. Pediatr Res. (1994) 36:159–68. 10.1203/00006450-199408000-00005 7970929

