# Supplementary material for: Mint Oil, ɤ-Tocopherol, and Whole Yeast Cell in Sow Diets Enhance Offspring Performance in the Postweaning Period
Source: Front Vet Sci. 2021 Jul 12;8:658956. doi: 10.3389/fvets.2021.658956 (PMC8311125; doi:10.3389/fvets.2021.658956)
Supplement: Supplementary file 1 [file Table_1.docx]

Supplementary Material

**Table 1.**Sow body weight (BW), backfat, and lactation feed intake provided a control diet, and diets with yeast cell (YC), ɣ-tocopherol (GT), and (MO) top dress in gestation and lactation.

| **Items** |  |  |  |  | **SEM** | **P-value** |
| --- | --- | --- | --- | --- | --- | --- |
|  | Study 1 | | | |  |  |
|  | **CON** | **YC** | **MO** | **YCMO** |  |  |
| No. sows | 12^1^ | 11 | 11 | 11^1^ |  |  |
| Parity distribution |  |  |  |  |  |  |
| P0 (gilt) | 4 | 4 | 4 | 4 |  |  |
| P1 | 0 | 1 | 0 | 0 |  |  |
| P2 | 7 | 5 | 6 | 6 |  |  |
| P3 | 1 | 1 | 1 | 1 |  |  |
| Sow BW, kg |  |  |  |  |  |  |
| D 110 of Gestation | 242.5 | 239.1 | 244.6 | 246.1 | 6.3 | 0.834 |
| Farrowing^2^ | 225.8 | 222.5 | 219.6 | 229.0 | 7.4 | 0.779 |
| Weaning | 226.4 | 213.9 | 207.6 | 215.5 | 12.8 | 0.673 |
| Sow backfat, mm |  |  |  |  |  |  |
| Entry | 12.9^x,y^ | 12.4^y^ | 15.3^x,y^ | 15.5^x^ | 1.0 | 0.031 |
| Wean | 11.1^x,y^ | 10.4^y^ | 12.2^x,y^ | 13.6^x^ | 0.91 | 0.085 |
| Lactation feed intake, kg/d | 6.41 | 6.59 | 6.68 | 6.64 | 0.42 | 0.952 |
|  |  |  |  |  |  |  |
|  | Study 2 | | | |  |  |
|  | **CON** | **YC** | **MO** | **GT** |  |  |
| No. sows | 13 | 14^3^ | 12 | 14 |  |  |
| Parity distribution |  |  |  |  |  |  |
| P0 (gilt) | 3 | 3 | 1 | 3 |  |  |
| P1 | 1 | 1 | 3 | 1 |  |  |
| P2 | 2 | 2 | 1 | 3 |  |  |
| P3 | 3 | 2 | 2 | 1 |  |  |
| P4 | 2 | 4 | 1 | 5 |  |  |
| P5 | 2 | 2 | 4 | 1 |  |  |
| Sow BW, kg |  |  |  |  |  |  |
| Breeding | 203 | 190 | 214 | 208 | 12.80 | 0.583 |
| Weaning | 224 | 228 | 233 | 228 | 7.05 | 0.832 |
| Sow back fat, mm |  |  |  |  |  |  |
| Breeding | 17.4 | 17.3 | 16.8 | 17.6 | 0.61 | 0.797 |
| D 110 of Gestation | 19.0 | 20.3 | 18.4 | 20.0 | 1.01 | 0.394 |
| Wean | 16.8 | 19.1 | 16.6 | 18.0 | 1.38 | 0.357 |
| Gestation feed intake, kg/d | 2.07^x,y^ | 2.03^y^ | 2.22^x,y^ | 2.22^x^ | 0.06 | 0.029 |
| Lactation feed intake, kg/d | 6.50 | 6.71 | 6.95 | 6.80 | 0.23 | 0.558 |

^1^One sow in each of CON and YMO and their litters were removed from the trial due to feed consumption issues in lactation and refusal to nurse piglets.

^2^Females weighed within 24h of parturition.

^3^One sow from the YC-fed group was euthanized during farrowing due to a twisted uterus.

^4^Values denoted with superscript ^x, y^indicates a tendency (*P*< 0.10).

^5^Values denoted with superscript ^a,b,c,d^ indicates a significance (*P*< 0.05).
